# Covid-19 triage in the emergency department 2.0: how analytics and AI transform a human-made algorithm for the prediction of clinical pathways

**DOI:** 10.1007/s10729-023-09647-2

**Published:** 2023-07-10

**Authors:** Christina C. Bartenschlager, Milena Grieger, Johanna Erber, Tobias Neidel, Stefan Borgmann, Jörg J. Vehreschild, Markus Steinbrecher, Siegbert Rieg, Melanie Stecher, Christine Dhillon, Maria M. Ruethrich, Carolin E. M. Jakob, Martin Hower, Axel R. Heller, Maria Vehreschild, Christoph Wyen, Helmut Messmann, Christiane Piepel, Jens O. Brunner, Frank Hanses, Christoph Römmele, on behalf of the LEOSS study group, Christoph Spinner, Maria Madeleine Ruethrich, Julia Lanznaster, Christoph Römmele, Kai Wille, Lukas Tometten, Sebastian Dolff, Michael von Bergwelt-Baildon, Uta Merle, Katja Rothfuss, Nora Isberner, Norma Jung, Siri Göpel, Juergen vom Dahl, Christian Degenhardt, Richard Strauss, Beate Gruener, Lukas Eberwein, Kerstin Hellwig, Dominic Rauschning, Mark Neufang, Timm Westhoff, Claudia Raichle, Murat Akova, Bjoern-Erik Jensen, Joerg Schubert, Stephan Grunwald, Anette Friedrichs, Janina Trauth, Katja de With, Wolfgang Guggemos, Jan Kielstein, David Heigener, Philipp Markart, Robert Bals, Sven Stieglitz, Ingo Voigt, Jorg Taubel, Milena Milovanovic

**Affiliations:** 1 Health Care Operations/Health Information Management, Faculty of Business and Economics, Faculty of Medicine, University of Augsburg, Universitätsstraße 16, 86159 Augsburg, Germany; 2 Professor of Applied Data Science in Health Care, Nürnberg School of Health, Ohm University of Applied Sciences Nuremberg, Nuremberg, Germany; 3 Anaesthesiology and Operative Intensive Care Medicine, Faculty of Medicine, University of Augsburg, Stenglinstrasse 2, 86156 Augsburg, Germany; 4 Department of Internal Medicine II, Technical University of Munich, School of Medicine, University Hospital Rechts Der Isar, Munich, Germany; 5 Hygiene and Infectiology, Klinikum Ingolstadt, Ingolstadt, Germany; 6 Department of Internal Medicine, Hematology and Oncology, Goethe University Frankfurt, Frankfurt Am Main, Germany; 7 Department I of Internal Medicine, University of Cologne, University Hospital of Cologne, Cologne, Germany; 8 German Center for Infection Research, Partner Site Bonn-Cologne, Cologne, Germany; 9 Clinic for Internal Medicine III - Gastroenterology and Infectious Diseases, University Hospital Augsburg, Stenglinstraße 2, 86156 Augsburg, Germany; 10 Clinic for Internal Medicine II - Infectiology, University Hospital Freiburg, Freiburg, Germany; 11 COVID-19 Task Force, University Hospital Augsburg, Stenglinstraße 2, 86156 Augsburg, Germany; 12 Hematology and Internal Oncology, University Hospital Jena, Jena, Germany; 13 Pneumology, Infectiology and Internal Intensive Care Medicine, Klinikum Dortmund, Germany; 14 Department of Internal Medicine, Infectious Diseases, University Hospital Frankfurt, Goethe University Frankfurt, Frankfurt Am Main, Germany; 15 Praxis am Ebertplatz, Cologne, Germany; 16 Department of Medicine I, University Hospital of Cologne, Cologne, Germany; 17 Department of Hemato-Oncology and Infectious Diseases, Klinikum Bremen-Mitte, Bremen, Germany; 18 Department of Technology, Management, and Economics, Technical University of Denmark, Hovedstaden, Denmark; 19 Data and Development Support, Region Zealand, Denmark; 20 Internal Medicine and Infectious Diseases, University Hospital Regensburg, Regensburg, Germany

**Keywords:** Covid-19 triage, Clinical decision making, Predictive analytics, Artificial intelligence, Machine learning

## Abstract

The Covid-19 pandemic has pushed many hospitals to their capacity limits. Therefore, a triage of patients has been discussed controversially primarily through an ethical perspective. The term triage contains many aspects such as urgency of treatment, severity of the disease and pre-existing conditions, access to critical care, or the classification of patients regarding subsequent clinical pathways starting from the emergency department. The determination of the pathways is important not only for patient care, but also for capacity planning in hospitals. We examine the performance of a human-made triage algorithm for clinical pathways which is considered a guideline for emergency departments in Germany based on a large multicenter dataset with over 4,000 European Covid-19 patients from the LEOSS registry. We find an accuracy of 28 percent and approximately 15 percent sensitivity for the ward class. The results serve as a benchmark for our extensions including an additional category of palliative care as a new label, analytics, AI, XAI, and interactive techniques. We find significant potential of analytics and AI in Covid-19 triage regarding accuracy, sensitivity, and other performance metrics whilst our interactive human-AI algorithm shows superior performance with approximately 73 percent accuracy and up to 76 percent sensitivity. The results are independent of the data preparation process regarding the imputation of missing values or grouping of comorbidities. In addition, we find that the consideration of an additional label palliative care does not improve the results.

**Supplementary Information:**

The online version contains supplementary material available at 10.1007/s10729-023-09647-2.

## Highlights

- We are the first to evaluate an existing triage algorithm for clinical pathways applied in German hospitals based on a unique German multicenter dataset.
- We propose analytics and AI-based extensions which improve performance metrics compared to those of the existing algorithm.
- We explicitly include the explainable AI discussion in literature and integrate explainable and easy-to-apply new approaches, as well.
- We study the influence of varying AI and non-AI data preparation strategies.

## Introduction

The Covid-19 pandemic has pushed many hospitals to their capacity limits. Therefore, triage of patients has been discussed controversially primarily through an ethical perspective (see, e.g., [28] or [30]. Even though the term triage seems to have taken on a weighty meaning with the pandemic, it is still not new and triage algorithms have been used for a long time like in the emergency department or for mass casualty incidents. Triage within mass casualty incidents is about saving as many patients as possible with limited resources outside the hospital [21, 37, 38]. For emergency departments, the task is on classifying arriving patients due to urgency of treatment whereby scarce resources play a subordinate role [17].

In Germany, physicians have not been forced to decide in view of scarce resources during the Covid-19 pandemic so far. But in general, Covid-19 triage with limited personnel and ventilation resources in hospitals integrates both approaches, i.e., emergency department and mass casualty incidents triage, and contains many aspects, such as urgency of treatment (e.g., [46], testing (e.g., [10]), severity of the disease (e.g., [11], access to critical care (e.g., [44] or the classification of patients regarding clinical pathways [39]. The classification of patients regarding clinical pathways determines ward, Intensive Care Unit (ICU), and outpatients starting from the emergency department. Although this classification is highly important for patient care and capacity planning in hospitals, it is hardly discussed in literature (see, e.g., reviews by [31, 47] or [1]. Regarding Covid-19 diagnosis (e.g., [48]), prognosis (e.g., [2] or [8] or [7], scores (e.g., [24] or [34], severity (e.g., [29] or mortality (e.g., [40], plenty of research has been proposed with a strong focus on Artificial Intelligence (AI) approaches. Symptoms, vital signs, medical imaging techniques, risk factors, blood counts or a combination of the categories are among the most integrated input parameters for the predictions (e.g., [5, 14, 35, 46, 47, 49]. The focus here is often on data-driven training and evaluation of standard models, without considering the actual application and transparency. In addition, it is noticeable that a broad data basis and the validation are usually lacking [47].

In this work, we evaluate the performance of triage algorithms for the classification of patients regarding clinical pathways based on a multicenter dataset with more than 4,000 Covid-19 patients of the Lean European Open Survey on COVID-19 Patients (LEOSS) registry. Compared with previous work, the size of our dataset significantly exceeds current literature [1]. The decision tree proposed by Pin et al. [39] is suggested by the German Society for Interdisciplinary Emergency and Acute Medicine (DGINA) to be considered as a guideline and applied in emergency departments in Germany, e.g., in the University Hospital of Augsburg. The results on the decision tree by Pin et al. [39] serve as a benchmark for our data-driven, AI and interactive human-AI extensions. Besides a base classifier regarding outpatient, ward, and ICU care, we research a hypothetical extension with outpatient, ward, ICU, and palliative care (i.e., death), to juxtapose data-induced and ethical considerations. As data issues arise in such settings, we study the influence of varying AI and non-AI data preparation strategies as well. We thus aim to close the validation and the application gap on a broad data basis for predicting the clinical course of incoming patients, which has not been in the focus of Covid-19 triage researchers so far. In addition, we take up the broad, ethical, and explainable AI (XAI) discussion in literature (see, e.g., [3] and present the performance of a human-AI interaction on the classification problem. We find significant potential of Covid-19 triage in the emergency department regarding accuracy, sensitivity, and other performance metrics. Comparing AI methods with the human-AI interaction, the human-AI approach shows similar performance in general and is significantly better at classifying ICU patients. An additional label palliative care does not improve the outcome, which is an important finding for the ethical discussion on Covid-19 triage.

Our work is structured as follows. In Section 2, we discuss the definitions and the literature which lay the basis for our methodology. Section 3 describes the data preparation process, the base triage algorithm, its data-driven extension, our AI systems, and performance metrics. Section 4 provides the results for both, a basic pathway classifier involving three labels (outpatient, ward, ICU) and an extended version with four labels (outpatient, ward, ICU, palliative care). The results are critically discussed in Section 5. Section 6 presents concluding remarks.

## Related definitions and literature

The healthcare sector faces substantial challenges such as staff shortages and increasing treatments, for which advances in digitalization are generally known as a possible solution. The Covid-19 pandemic has aggravated the problem of staff shortages. Artificial Intelligence and Machine Learning are an important base for digitalization in healthcare. Often and in this work as well the terms are used synonymously, but in fact Machine Learning is defined as a part of Artificial Intelligence [19]. While Artificial Intelligence focuses on autonomous algorithmic decisions in general, Machine Learning denotes a machine autonomously learning from data. There exist supervised and unsupervised Machine Learning methods. Unsupervised methods aim at clustering of unlabeled data and supervised Machine Learning methods focus on classification and regression problems for labeled data.

Machine Learning methods such as decision tree, Multilayer Perceptron (MLP), Extreme Gradient Boosting (XGB) or Random Forest (RF) are attributed to the category of predictive analytics. Predictive analytics summarize different approaches for event prediction. Descriptive analytics summarize different statistical approaches for the descriptive and retrospective analysis of data. Prescriptive analytics aim at prospective decision support based on statistical and mathematical programming techniques [32].

We use analytics as a general term for mathematical and statistical methods with the aim to learn from data and focus on a classification problem in a Covid-19 setting. The (meta-) pathway of incoming Covid-19 patients starting from the emergency department is to be determined. Patients are assigned to the ward unit, the intensive care unit (ICU), the palliative care unit (PCU) or are discharged, i.e., outpatient, from the hospital. Our four different (meta-) pathways are defined as follows: (1) ED $\to$ Discharge, (2) ED $\to$ Ward, (3) ED $\to$ ICU, and (4) ED $\to$ PCU. By the determination of the pathway, an incoming Covid-19 patient is triaged with respect to the subsequent place of treatment. In times of digitalization in healthcare, the question is not only how analytics influence the decision-making process, but also the question remains as to who is making the actual triage decision of patients. The triage can be done autonomously by a physician experienced in Covid-19 care, i.e., human approach, autonomously by a supervised Machine Learning algorithm trained with relevant data, i.e., AI approach, or any interactive version of the options, i.e., interactive human-AI approach (see detailed definition on human-AI interaction below).

As the concept of triage itself raises ethical concerns because patients are grouped with potential consequences for their well-being, so does AI-based decision support. Various requirements for an ethical AI have been elaborated in literature (see, e.g., [36, 45] or [6]. The requirements include, among others, the autonomy of physicians and patients or a certain transparency of the methods. The definition of this transparency of AI methods is controversial in the literature stream on Explainable Artificial Intelligence (XAI). Arrieta et al. [3] define transparency, i.e., interpretability, as an intrinsic characteristic of a model. For example, decision trees are referred to transparent methods, because the structure and decision-making process immediately become visually clear to the user. According to Arrieta et al. [3], explainability is an extrinsic characteristic of a model. Multilayer Perceptron or Random Forest, for instance, are non-transparent models with a certain potential for explainability, because simplification techniques or feature importance analyses might contribute to explainability for the user. Understandability is to be distinguished from transparency and explainability according to Arrieta et al. [3]. An algorithm is defined to be understandable, if and only if the algorithmic decision is understandable. The major goal of XAI is trustworthiness in the AI-based models, which is a basic prerequisite, among technological concerns, for the actual use of the techniques in healthcare institutions. Fuhrman et al. [18] apply a similar distinction of explainability and transparency, i.e., interpretability, in their review on AI-assisted medical imaging in Covid-19 settings, while Tjoa and Guan [41] use the terms explainability and transparency synonymously. Tjoa and Guan [41] do not distinguish between intrinsic and extrinsic characteristics but concentrate on the efforts to make the algorithmic decision transparent to the user. In this work, we differentiate between explainability and transparency, i.e., interpretability, as Arrieta et al. [3] or Fuhrman et al. [18] do. The term XAI is used as a general term defining the research stream of ensuring trustworthy AI-based decisions.

Human-AI interaction might contribute to XAI in healthcare [22]. Van Berkel et al. [42] generally “define human-AI interaction as the completion of a user’s task with the help of AI support […]” and describe the wide variety of different human-AI interactions with respect to the initiator of the interaction, the timepoints of the interaction in the decision-making process, and the user’s reaction. For example, an AI-based clinical decision support system might suggest a certain classification of a patient. The interaction might be the system’s output which is the basis for the classification of the patient by a physician or consecutive decision-making depending on the predicted outcome or any other interactive decision-making process.

Not only transparency and interaction influence the actual application of decision support in hospitals, but also implementation issues and usability. As there are different advances in many countries regarding digitalization in hospitals, e.g., the Hospital Future Act in Germany,^1^ the broad implementation of digital decision support tools will be made possible in the near future. Reviews on the usability of mobile apps and mobile health apps can be found in Harrison et al. [20] and Azad-Khaneghah et al. [4]. Usability is mainly determined by “Effectiveness”, “Efficiency” and “Satisfaction” [23] of the application and is strongly associated with the performance, transparency, and implementation of the algorithm, consequently.

We assess the influence of the decision maker, the analytics-based definition, and the transparency of the decision-making process on the accuracy of Covid-19 triage in the emergency department. To evaluate the influence, four different approaches are examined: the base triage algorithm (TA) proposed by Pin et al. [39], an analytics-based extended version of the base algorithm (TAE), AI-based algorithms, and an integrated triage algorithm (ITA). The four approaches vary in the decision maker, the definition, and the transparency of the decision-making process (see Table 1). For the AI-based algorithms, we apply Multilayer Perceptron (MLP), Extreme Gradient Boosting (XGB), and Random Forest (RF). We take a data driven retrospective perspective which lays the basis for a prospective evaluation, and the ethical discussion about AI-based decision support for Covid-19 triage in the emergency department. In addition, we aim at a contribution to the discussion on the ethics of triage by evaluating the flexible inclusion of a palliative care label in some algorithms.

**Table 1:** Comparison of the different approaches for Covid-19 triage in the emergency department

Determinant		TA	TAE	AI	ITA
1	Decision maker	Human	Human	Machine	Interactive
2	Analytics-based definition of the decision-making process	No	Yes	Yes	Yes
3	Transparency of the decision-making process	Yes	Yes	No	Partly
4	Flexible inclusion of a palliative care label	No	Yes	Yes	No

## Methods

### Data processing

Our study is based on a LEOSS data export with 4,310 Covid-19 patients and 190 columns (i.e., features) from January 2021. Thus, our study captures the first and second pandemic wave in Europe (March 18, 2020, with January 7, 2021). The Lean European Open Survey on SARS-CoV‑2 infected Patients project is a prospective European multi-center cohort study that enables retrospective analyses on a broad basis [26]. We consider LEOSS baseline data due to our interest in parameters collected at an early stage of infection. In the LEOSS protocol, diagnosis is confirmed via PCR or rapid tests as an acceptable alternative. To ensure anonymity in all steps of the analysis process, an individual LEOSS Scientific Use File was created, which is based on the LEOSS Public Use File principles described in Jakob et al. [25]. The study was conducted in accordance with the Declaration of Helsinki Ethical Principles and Good Clinical Practices and was reported to the local Ethics Committee.

The raw data contains demographical features, blood counts, vital signs, Covid-19 related symptoms, comorbidities, medical imaging outcomes and the clinical (meta-) pathway of the patients. First, the raw data was cleaned up regarding incorrect entries. Data preparation for the remaining data set is based on feature importance (e.g., vital signs and laboratory parameters) or commonly known methodologies (percentage of blank rows). Since statistical guidelines recommend using data with more than 40 percent missing entries solely as hypothesis generation, these columns are removed beforehand (e.g., [16, 27]. Furthermore, vital signs and laboratory parameters have a high impact on the course of Covid-19 disease, which is why at least two values of them must be filled in. In general, missing values are a common problem in healthcare. In order not to ignore any highly relevant features, the removed features were discussed with experts. In addition, the remaining missing values need to be filled since not all machine learning algorithms and oversampling techniques (see below) are able to handle missing values. The methods used for filling in empty values include a simple imputer and two iterative machine learning imputers (Random Forest and Multi-Layer-Perceptron algorithms). Following the creation of the three different datasets by filling in the empty values, the comorbidities are summarized. This is a common procedure in the Covid-19 literature to reduce complexity while retaining important information. There are two different variants for the summary of comorbidities, namely the sum of the comorbidities and the Charlson Comorbidity Index [13].

The different data preparations were divided into feature and label matrices. Our label definition leads to two different formats, as we distinguish between three (3) and four (4) (meta-) pathways in the following: The base case with ED $\to$ Discharge (i.e., outpatient), ED $\to$ Ward (i.e., ward), and ED $\to$ ICU (i.e., ICU), may be extended by a palliative care label (ED $\to$ PCU) which has been incorporated in the base case labels before. All patients who were in the ICU (or Intermediate Care, IMC) during their hospital stay were assigned to the ED $\to$ ICU pathway (i.e., ICU), all other inpatients to the ED $\to$ Ward (i.e., ward), pathway, and the rest to the ED $\to$ Discharge (i.e., outpatient) pathway. In the four-label classification, all deceased patients were assigned to the ED $\to$ PCU (i.e., palliative care) pathway. Please find a detailed description of our data preparation and label definition in Supplementary Fig. 1.

Depending on the imputer, the summary of comorbidities, and the label definition, we define twelve different input data sets with 3,543 patients and 58 features each: six for each of the two different classifications with three or four labels, whereby three different imputers (Simple Imputer, RF, MLP) and two different summaries of comorbidities (Sum, CCI) are applied. Table 2 provides an overview of the twelve different input data sets. Table 3 lists the 58 different features per input data set.

**Table 2:** Description of the 12 different input data sets (RF: Random Forest, MLP: Multiple Layer Perceptron, CCI: Charlson Comorbidity Index)

ID	Data set	Number of labels	Imputer	Comorbidities
1	3RC	3	RF	CCI
2	3RS	3	RF	Sum
3	3MC	3	MLP	CCI
4	3MS	3	MLP	Sum
5	3SC	3	Simple imputer	CCI
6	3SS	3	Simple imputer	Sum
7	4RC	4	RF	CCI
8	4RS	4	RF	Sum
9	4MC	4	MLP	CCI
10	4MS	4	MLP	Sum
11	4SC	4	Simple imputer	CCI
12	4SS	4	Simple imputer	Sum

**Table 3:** Description of the 58 features in the input data sets (CT: Computer tomography, CCI: Charlson Comorbidity Index)

No	Feature
1	Age
2	Gender
3	At least one neuronal disease (binary)
4	At least one cardiovascular disease (binary)
5	Prior heart failure
6	Stage heart failure
7	BMI: Body Mass Index
8	Asymptomatic symptoms
9	Runny nose
10	Sore throat
11	Dry cough
12	Productive cough
13	Wheezing
14	Dyspnoe
15	Palpitations
16	Diarrhea
17	Nausea / emesis
18	Muscle aches
19	Muscle weakness
20	Fever
21	Delirium
22	Excessive tiredness
23	Headache
24	Meningism
25	Smell disorder
26	Taste disorder
27	Other neurological findings
28	Red eye
29	Systolic blood pressure
30	Diastolic blood pressure
31	Pulse
32	Respiratory rate
33	sO2: Oxygen saturation
34	Temperature
35	CT: Air trapping
36	CT: Areas of consolidation
37	CT: Bronchiolitis
38	CT: Crazy paving pattern
39	CT: Ground glass opacities
40	CT: Interlobular septal thickening
41	CT: Nodulary lesions
42	CT: Pleural effusion
43	Other relevant CT results
44	AST: Aspartate transaminase
45	ALT: Alanine transaminase
46	GGT: Gamma-glutamyl transferase
47	Bilirubine
48	Creatinine
49	Urea
50	LDH: Lactate dehydrogenase
51	D-dimer
52	Leukocytes
53	Lymphocytes
54	Neutrophils
55	Platelets
56	Hemoglobin
57	CRP: C-reactive protein
58	CCI / Sum

To avoid overfitting throughout our study, we used ten-fold cross validation. Each input data set is randomly split into ten different folds, while every subset is subsequently defined as test data set with a training and testing ratio of 90% and 10%. Performance is then measured based on the metrics for the different test data sets.

### Base triage algorithm and data-based extension

**Fig. 1:**
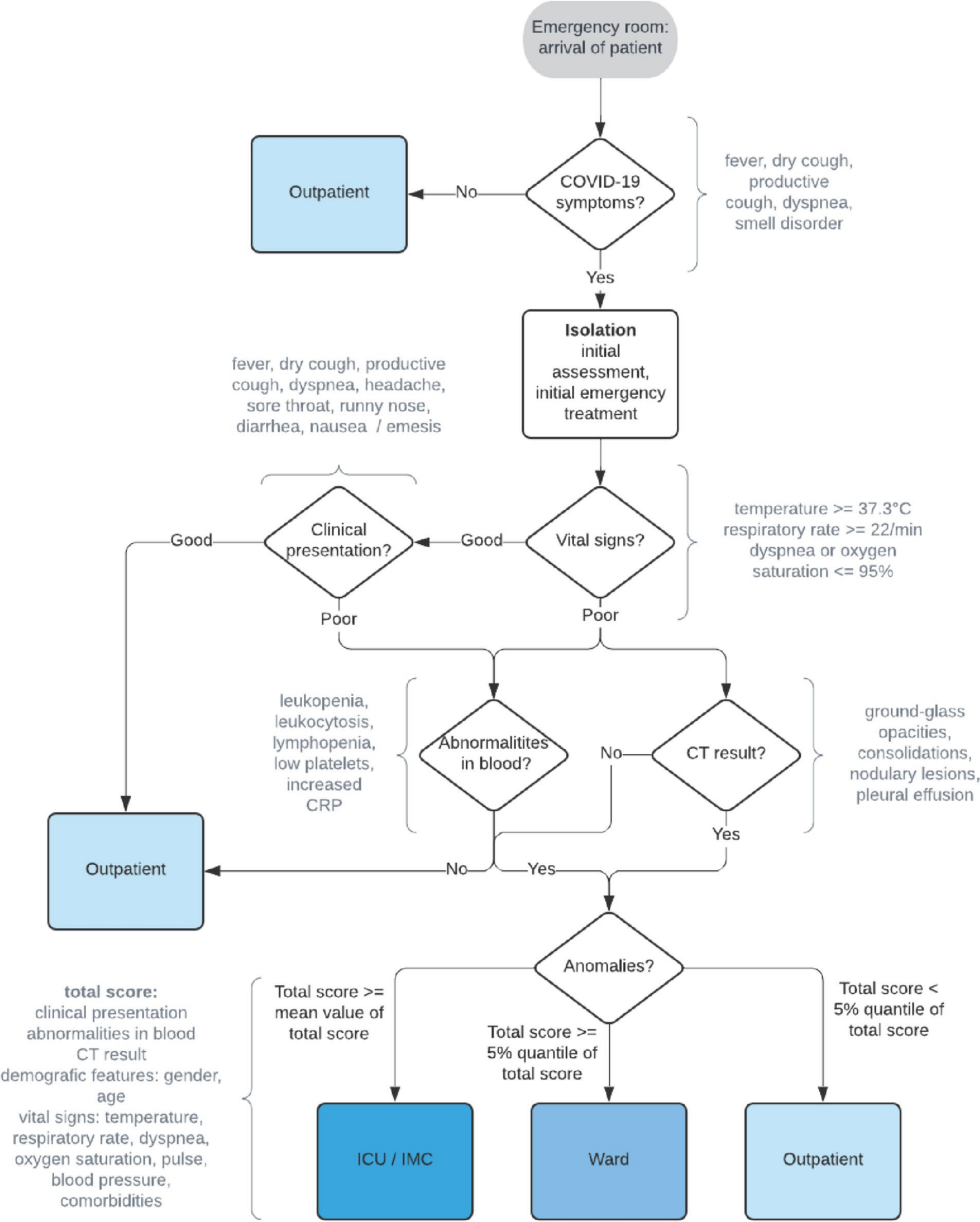
Base triage algorithm (TA) according to Pin et al. [39]

Figure 1 illustrates the base triage algorithm (TA) for clinical pathways of Covid-19 subjects [39] which is considered as a guideline for emergency departments in Germany as suggested by DGINA. TA is constructed as an easy-to-understand and simply applicable decision tree. After examining classical Covid-19 symptoms (such as dry cough and vital signs), the overall clinical presentation are evaluated. Step three involves blood counts and medical imaging. Finally, the results of all steps of the algorithm are reviewed in their entirety and the patient is classified as outpatient (i.e., discharge), ward, or ICU. Other than for ED $\to$ Ward and ED $\to$ ICU, a physician can also classify the pathway ED $\to$ Discharge based on steps one to three. Note, TA only involves three classification labels with outpatient (i.e., discharge), ward, or ICU.

**Fig. 2:**
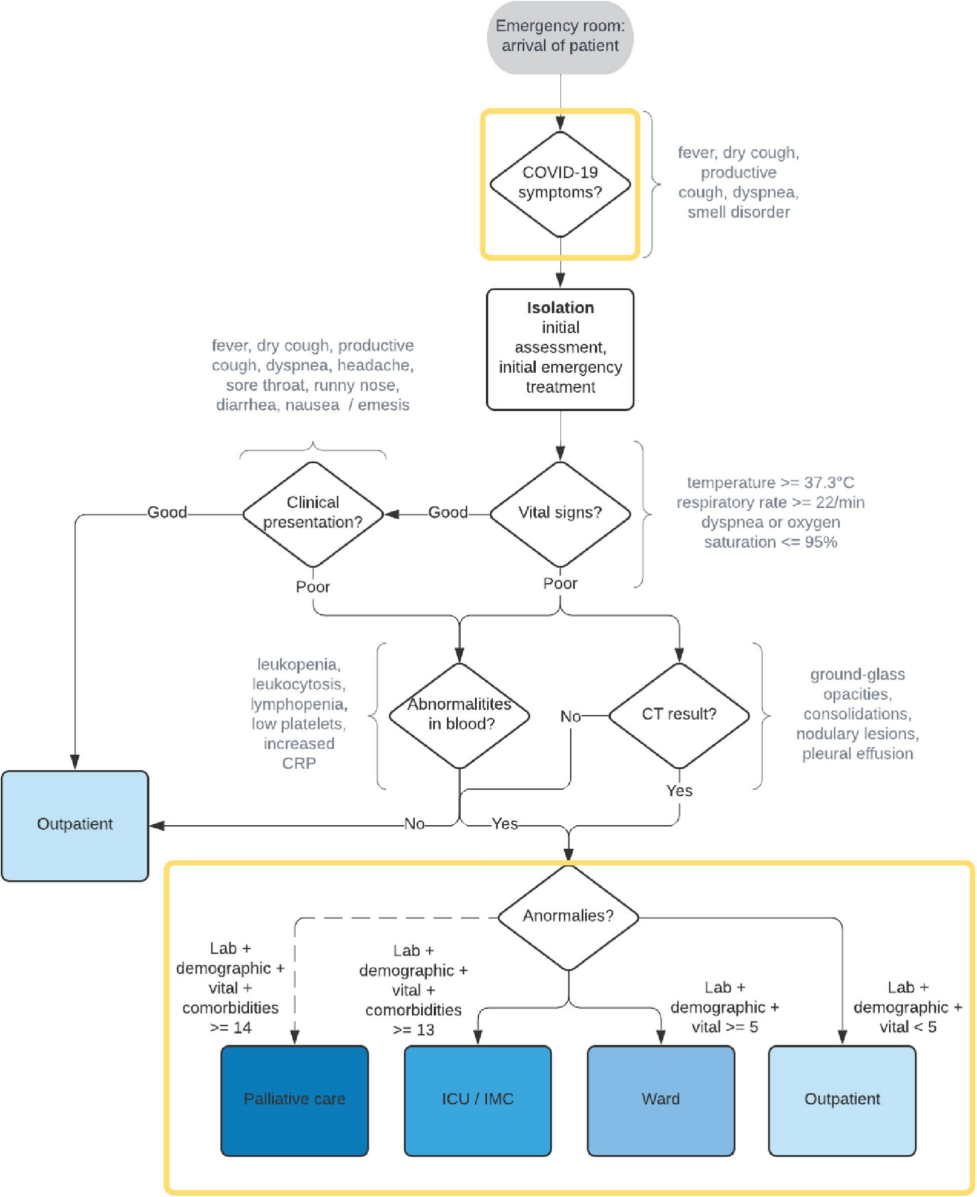
Extended triage algorithm (TAE). Yellow boxes highlight the differences compared to TA (see Fig. 1). TAE Scores for laboratory values, vital signs, demographic values, and comorbidities are shown in Table 4

Our extended version of the base algorithm (TAE) flexibly considers either three labels or an optional fourth classification label (i.e., palliative care), while TAE always builds upon alternative analytics-based first and final steps (see Fig. 2). The new first step in TAE avoids the discharge of patients (i.e., ED $\to$ Discharge) in the first step of the algorithm. Due to a significant number of asymptomatic patients in the data, the finding of a symptom-free status may not be sufficient to classify the patient as an outpatient. Therefore, in contrast to TA, patients arriving in the emergency department always have their vital signs and clinic checked after symptoms were reviewed. The new final step, namely the calculation of the TAE score, is based on findings of abnormalities and risk factors for severe Covid-19 progression in literature (e.g., [15, 33, 43]). Compared to the TA, the TAE score includes the severity of an anomaly. For example, a distinction is made among the laboratory values as to whether a patient's temperature is only elevated or high. Together, these form a TAE score to classify patients with high accuracy (see Table 4). Both implemented changes compared to the TA are highlighted with yellow boxes in Fig. 2.

**Table 4:** TAE Scores for lab, vital, demographics, comorbidities

Lab		Vital		Demographics		Comorbidities	
Lymphocytes		Temperature		Age		Sum	
*500—1499 /µL*	+ *1*	*37.3—37.9 °C*	+ *1*	*46—55*	+ *1*	$\ge$*1*	+ *1*
*100—499 /µL*	+ *3*	*38.0—39.9 °C*	+ *2*	*56—65*	+ *2*	$\ge$*2*	+ *2*
< *100 /µL*	+ *4*	> *39.9 °C*	+ *3*	*66—75*	+ *3*	CCI	
Leukocytes		sO2		> *76*	+ *4*	$\ge$*0.12*	+ *1*
*12,000—19,999 /µL*	+ *1*	*80—95%*	+ *1*	Gender		$\ge$*0.26*	+ *2*
> = *20,000 /µL*	+ *2*	*70—79%*	+ *2*	*Male*	+ *1*		
*1,000—3999 /µL*	+ *2*	*60—69%*	+ *3*				
< *1,000 /µL*	+ *3*	< *60%*	+ *4*				
Platelets		Respiratory rate					
*50,000—119,999 /µL*	+ *1*	*22 – 29 / Min*	+ *1*				
*10,000—49,999 /µL*	+ *2*	> *29 / Min*	+ *2*				
< *10,000 /µL*	+ *3*	Hypertension	+ *1*				
LDH, D-Dimer							
> *ULN*	+ *1*						
> *2xULN—10xULN*	+ *2*						
> *10xULN*	+ *3*						

### AI and human-AI systems

We focus a classification modeling problem and thus apply a Multi-Layer Perceptron (MLP), a Random Forest (RF) and an Extreme Gradient Boosting (XGB) classifier to the data. The MLP is characterized by a multi-layer neural network structure consisting of an input layer, several hidden layers, and an output layer. The RF consolidates the predictions of different decision trees based on a majority decision. The XGB algorithm is also constructed from decision trees by an ensemble or boosting idea. Note that these AI approaches, while generating an autonomous classifier, are of a black-box style and do not, other than a (simple) decision tree, meet the transparency requirements by Arrieta et al. [3].

**Fig. 3:**
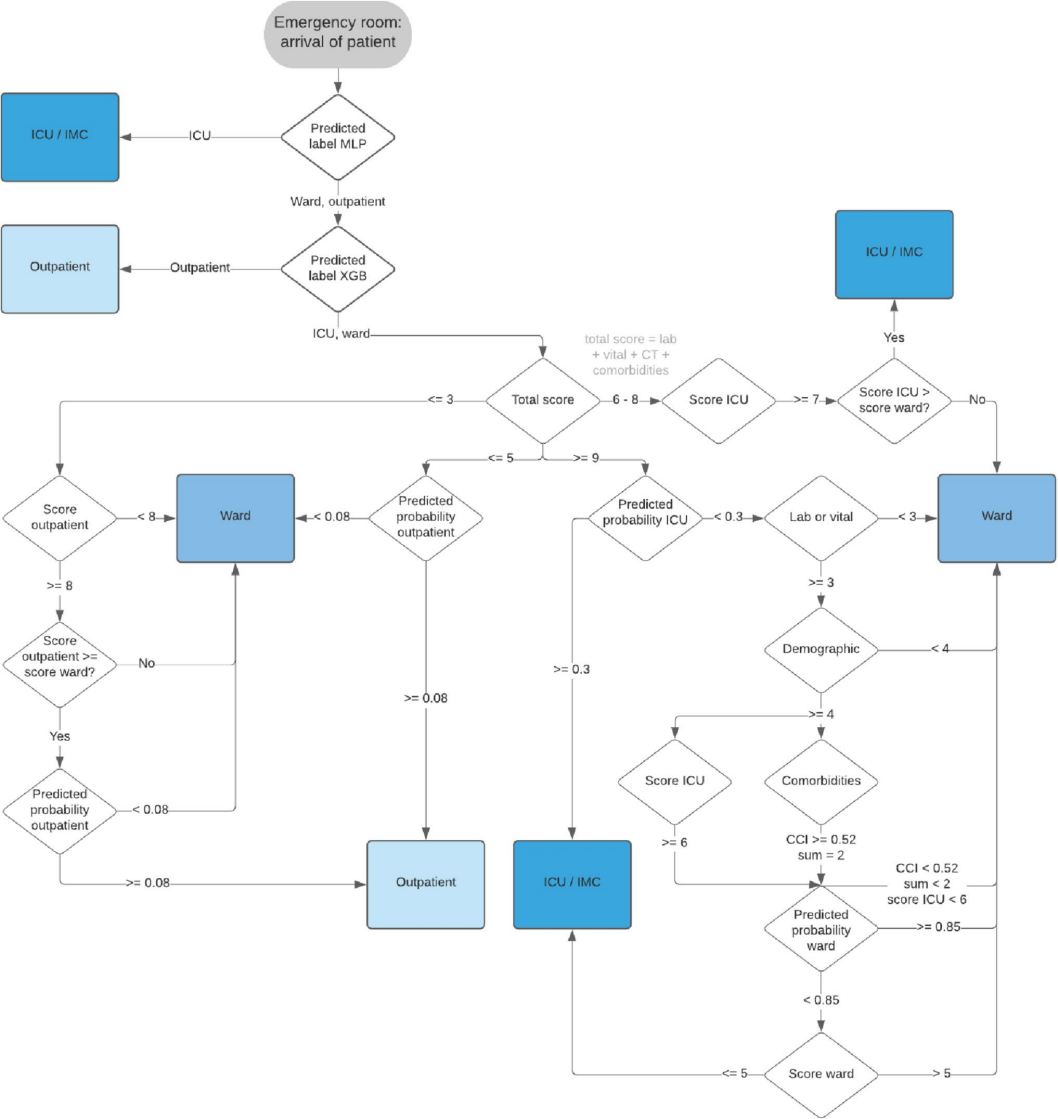
Integrated triage algorithm (ITA). ITA Scores for ICU, ward, and outpatient are shown in Table 5

In addition to the existing decision tree by Pin et al. ([39], TA) and the machine learning methods (MLP, RF, XGB), we investigate the potential of integrating both approaches in a two-step process: integrated triage algorithm (ITA). An AI-based autonomous pre-triage is made before the physician starts the actual triage of patients by means of a data-guided decision tree based on the ITA scores given in Table 5. The literature-based TAE scores (see Table 4) are incorporated into the recalculated ITA scores (see Table 5). In contrast to the TAE scores, scores for the different clinical pathways are formed in the ITA score. The calculation of the scores is based on feature importance, detailed data analytics, and discussions with experts. In the ITA algorithm, first, sequential MLP and XGB algorithms filter ICU patients and outpatients (i.e., discharge) based on the accurate prediction. Second, based on a white-box decision tree and the ITA scores, the remaining patients are classified as ICU, ward, or outpatient. By combining both approaches, we aim at the evaluation of a human-AI interactive algorithm, with autonomous black-box and white-box components. The autonomous pre-triage component (i.e., the black box model) saves working time of medical staff that has become scarce during the pandemic, while the second component (i.e., the white-box model) contributes to transparency. The two-step process, in addition, is of a human-AI interactive type because the pre-triage’s output is the basis for the classification of the patient by a physician. Fig. 3 presents our human-AI ITA algorithm.

**Table 5:** ITA Scores for ICU, ward, outpatient (TAE Scores are shown in Table 4)

ICU		Ward		Outpatient	
Avg pred. prob. ML ICU		Avg pred. prob. ML ward		Avg pred. prob. ML outpatient	
*0.2 – 0.59*	+ *1*	*0.4 – 0.59*	+ *1*	*0.2 – 0.59*	+ *1*
*0.6 – 0.89*	+ *2*	*0.6 – 0.89*	+ *2*	*0.6 – 0.89*	+ *2*
*0.9 – 1*	+ *3*	*0.9 – 1*	+ *3*	*0.9 – 1*	+ *3*
Lab		Lab		Lab	
*0 – 3*	+ *1*	*0 – 1;* > *11*	+ *1*	> *5*	+ *1*
*4 – 11*	+ *2*	*2 – 7*	+ *2*	*2 – 4*	+ *2*
> *11*	+ *3*	*8 – 11*	+ *3*	*0 – 1*	+ *3*
Vital		Vital		Vital	
*1 – 2*	+ *1*	*0 – 1;* > *9*	+ *1*	> *3*	+ *1*
*3 – 8*	+ *2*	*2 – 7*	+ *2*	*1.5 1 – 3*	+ *2*
> *8*	+ *3*	*8*	+ *3*	< *1.51*	+ *3*
Comorbidities		Comorbidities		Comorbidities	
*CCI*$\le$*0.26; Sum* = *1*	+ *1*	*CCI* = *0.52; Sum* = *2*	+ *1*	*CCI* = *0.26; Sum* = *2*	+ *1*
*CCI* = *0.52; Sum* = *2*	+ *2*	*CCI* = *0.26; Sum* = *1*	+ *2*	*CCI* = *0.12; Sum* = *1*	+ *2*
*CCI* = *0.85*	+ *3*	*CCI* = *0.85; CCI* = *0.12*	+ *3*	*CCI *$\ge$* 0.52*	+ *3*

### Performance measurement

We measure and compare the algorithms’ ability to correctly predict the patient (meta-) pathway in terms of outpatient (i.e., discharge), ward, ICU, and palliative care, by accuracy, sensitivity (i.e., recall), specificity, F1-score, precision, and the area under the receiver operating characteristic (ROC AUC).

While accuracy provides information on the correctly classified patients, precision focuses the true positive results divided by the positively classified. F1-score and ROC AUC incorporate either precision and sensitivity or specificity and sensitivity. The reported metrics are based on a ten-fold cross validation, hyperparameter tuning and the Synthetic Minority Oversampling Technique (SMOTE) to meet the problem of imbalanced data. SMOTE fills in the underrepresented classes in the data set by a resampling mode. Particularly in the case of multiclass classification, SMOTE achieves good results with respect to imbalanced data [9]. Hyperparameter tuning is a preprocessing optimization technique to the actual optimization of, for example, weights in a multi-layer neural network and defines hyperparameters such as the learning rate. Please note there exist different forms of AUC depending on the characteristics of the data set. Since our data set is balanced by SMOTE, we consider ROC AUC. However, in the case of imbalanced data it may be more appropriate to use a form of partial AUC as suggested by Carrington et al. [12]. A simple dummy classifier (DC) randomly classifying subjects as outpatient, ward, ICU, and palliative care patients with equal probability serves as a benchmark for the different classifiers.

## Results

In total, 3,543 Covid-19 patients are included in our study. Table 6 provides an overview on data availability, important demographic, and clinical characteristics of the patients. Most patients in the data set are over the age of 56 years old, male, and suffer from one cardiovascular disease at least. Fever is the most frequent classical Covid-19 symptom, followed by dry cough and dyspnea. Gamma-glutamyl transferase (GGT) and Lactat-dehydrogenase (LDH) are frequently increased in the patients. The distribution of labels is characterized by the fact that most patients in the data set remain in ward (see Table 7). Few patients are discharged from the hospital upon presentation in an emergency department.

**Table 6:** Demographic and clinical values at admission of COVID-19 patients

	Number of patients		Median category	Number of filled cells	
	Total	Pct		Total	Pct
Age			*56—65 years*	*3,527*	*99.55%*
< *1 years*	*10*	*0.28%*			
*1—3 years*	*11*	*0.31%*			
*4—8 years*	*6*	*0.17%*			
*9—14 years*	*7*	*0.20%*			
*15—17 years*	*0*	*0.00%*			
*18—25 years*	*72*	*2.04%*			
*15–25 years*	*20*	*0.57%*			
*26—35 years*	*229*	*6.49%*			
*36—45 years*	*311*	*8.82%*			
*46—55 years*	*535*	*15.17%*			
*56—65 years*	*676*	*19.17%*			
*66—75 years*	*605*	*17.15%*			
*76—85 years*	*768*	*21.77%*			
> *85 years*	*277*	*7.85%*			
Gender			*Male*	*3,543*	*100.00%*
*Male*	*2,094*	*59.10%*			
*Female*	*1,449*	*40.90%*			
At least one neuronal disease	*742*	*23.77%*	*No*	*3,122*	*88.12%*
At least one cardiovascular disease	*1,968*	*56.85%*	*Yes*	*3,462*	*97.71%*
Dry cough	*1,171*	*35.54%*	*No*	*3,295*	*93.00%*
Dyspnoe	*968*	*30.43%*	*No*	*3,181*	*89.78%*
Fever	*1,405*	*42.64%*	*No*	*3,295*	*93.00%*
Respiratory rate			*16—21*	*2,173*	*61.33%*
< *16*	*477*	*21.95%*			
*16—21*	*1,011*	*46.53%*			
*22—29*	*491*	*22.60%*			
> *29*	*194*	*8.93%*			
sO2			*90—95%*	*2,861*	*80.75%*
< *60%*	*26*	*0.91%*			
*60—69%*	*14*	*0.49%*			
*70—79%*	*67*	*2.34%*			
*80—89%*	*372*	*13.00%*			
*90—95%*	*1,130*	*39.50%*			
*96—100%*	*1,252*	*43.76%*			
Temperature			*37.3—37.9 °C*	*2,932*	*82.75%*
< *35.1 °C*	*12*	*0.41%*			
*35.1—37.2 °C*	*1,212*	*41.34%*			
*37.3—37.9 °C*	*630*	*21.49%*			
*38—38.9 °C*	*731*	*24.93%*			
*39—39.9 °C*	*298*	*10.16%*			
> *39.9 °C*	*49*	*1.67%*			
CT: Areas of consolidation	*369*	*16.01%*	*No*	*2,305*	*65.06%*
CT: Ground glass opacities	*578*	*25.08%*	*No*	*2,305*	*65.06%*
GGT			> *ULN*	*3,308*	*93.37%*
*Normal (LLN—ULN)*	*1,542*	*46.61%*			
> *ULN*	*522*	*15.78%*			
> *2* × *ULN*	*255*	*7.71%*			
> *5* × *ULN*	*83*	*2.51%*			
> *10* × *ULN*	*32*	*0.97%*			
< *LLN*	*874*	*26.42%*			
LDH			> *ULN*	*2,619*	*73.92%*
*Normal (LLN—ULN)*	*950*	*36.27%*			
> *ULN*	*1,347*	*51.43%*			
> *2* × *ULN*	*292*	*11.15%*			
> *5* × *ULN*	*16*	*0.61%*			
< *LLN*	*14*	*0.53%*			
*Lymphocytes*			*800 – 1,499 /µL*	*2,339*	*66.02%*
< *100 /µL*	*41*	*1.75%*			
*100—299 / µL*	*126*	*5.39%*			
*300—499 / µL*	*206*	*8.81%*			
*500—799 / µL*	*533*	*22.79%*			
*800—1,499 / µL*	*951*	*40.66%*			
*1,500—2,999 / µL*	*431*	*18.43%*			
> = *3,000 /µL*	*51*	*2.18%*

**Table 7:** Label distribution

No. of labels	Outpatient	Ward	ICU	Palliative care	Total
Three	*124*	*2,454*	*965*	-	*3,543*
Four	*117*	*2,209*	*625*	*592*	*3,543*

### Outpatient, ward, and ICU classifier: three labels

**Fig. 4:**
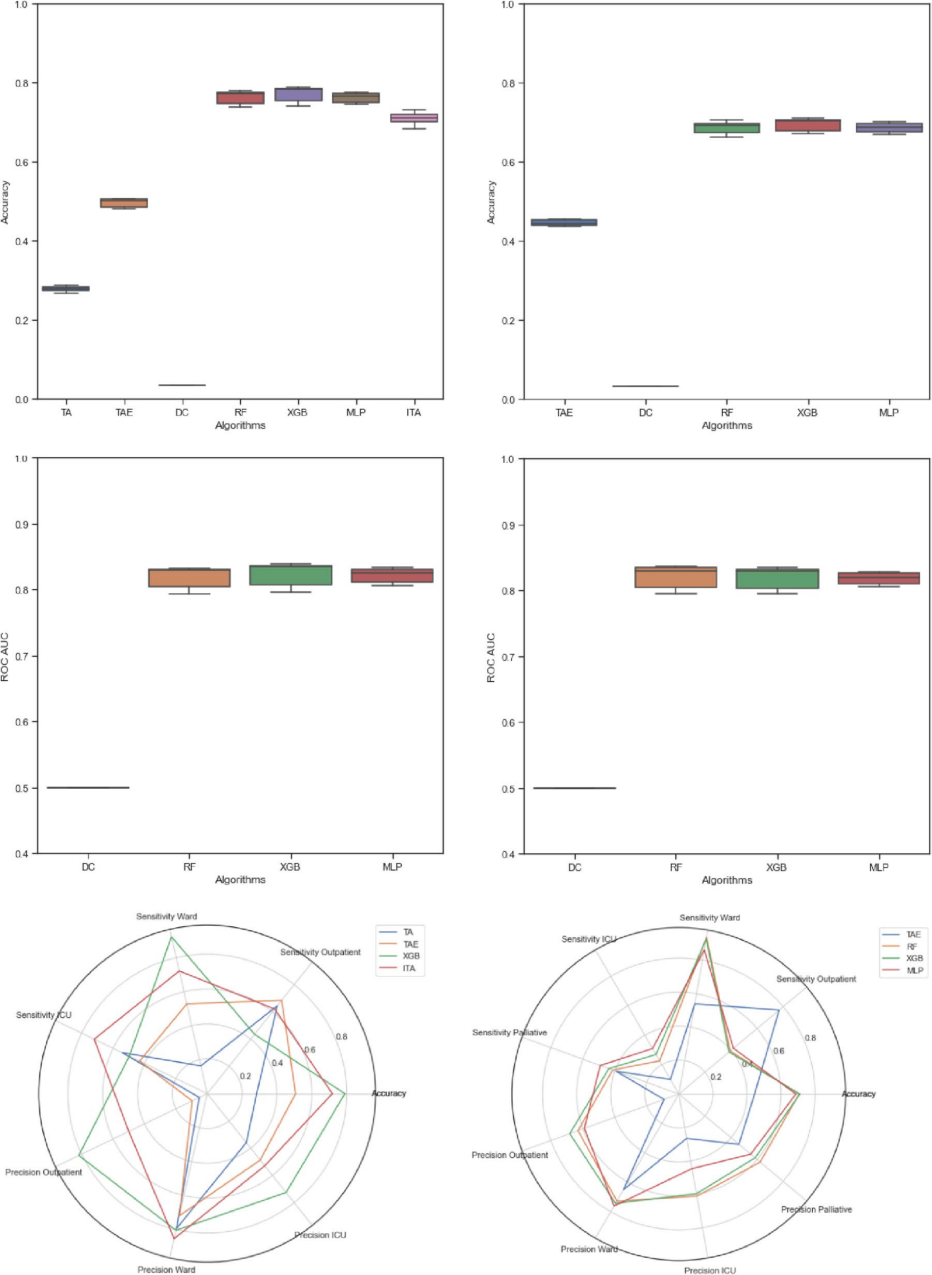
Comparison of the algorithms based on the accuracy (upper), ROC AUC (middle) and radar charts (lower) for data sets with 3 labels (left hand side) and four labels (right hand side). The respective boxplot represents the distribution of accuracy for the different data preparations. Both radar charts compare sensitivities, precision, and accuracies of the different algorithms. On the left-hand side, the XGB is used for all machine learning models, because of the similar performance

**Fig. 5:**
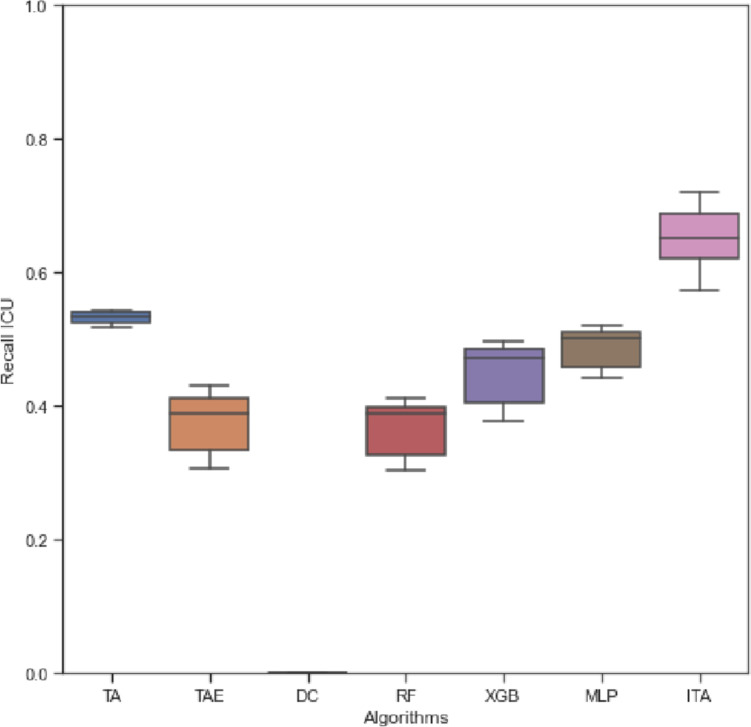
Comparison of the algorithms based on the recall of ICU. The respective boxplot represents the distribution of recalls for the different data preparations with 3 labels

In this section, we compare the base triage algorithm (TA), it’s extension (TAE), the dummy classifier (DC), the three machine learning techniques (MLP, RF, and XGB) and the integrated triage algorithm (ITA) for the base classifier task with three labels, outpatient, ward, and ICU. The overall accuracy ranges between 27% for TA, approx. 51% for TAE, approx. 73% for ITA, and up to 78% for the machine learning techniques (see Fig. 4). By comparison, the DC achieves only 4% total accuracy and a 50% ROC AUC. The machine learning algorithms obtained a significantly higher ROC AUC (between 76 and 88%, see Fig. 4). Differences are more in the labels than in the AI methods. The TA demonstrates high sensitivity for the outpatient class (up to 84%) but shows poor performance in classifying ward patients (approx. 15%). The TAE demonstrates a better performance regarding ward patients (up to 54%), while sensitivity in terms of the ward class is highest for the machine learning techniques (up to 94%). Regarding the ICU class, sensitivities vary from 43% (TAE) to 72% (ITA, see Fig. 5). While precision of TA varies significantly for the three labels (4% vs. 82%), the performance of MLP, RF and XGB is rather balanced here. Observing the specificity, it is noticeable that especially the ITA evokes rather balanced values between 63 and 98% compared to the ML algorithms (33% vs. 100%). The AI and human-AI methods consistently obtain higher F1-scores than the TA and TAE techniques. A detailed summary of the performance metrics provides Supplementary Table 1. A radar chart for a visual comparison of performance metrics for the three labels is provided in Fig. 4. The radar chart underlines the results of a poor performance of TA compared with the AI-based algorithms in all metrics. In addition, the significant improvement of the sensitivity for the ICU label and the ITA is illustrated.

In the synopsis of the results, AI and human-AI methods in most metrics outperform TA and TAE. Comparing the three machine learning classifiers (i.e., MLP, RF and XGB), XGB, a MLP imputer and the Charlson-Comorbidity Index for grouping comorbidities should be preferred. However, data processing has a minor influence on the performance metrics, overall. The confusion matrix and ROC AUC for the preferred XGB algorithm with three labels, a MLP imputer, and the Charlson-Comorbidity Index (i.e., 3MC data set) are presented in Supplementary Fig. 2.

### Outpatient, ward, ICU, and palliative care classifier: four labels

In case of four labels (i.e., outpatient, ward, ICU, palliative care), we compare the TAE, the DC, and the three machine learning techniques (MLP, RF and XGB). The overall accuracy decreases for TAE, MLP, RF, and XGB (see Fig. 4). Nonetheless, the basic statement remains that a significant improvement is achieved here by machine learning techniques. The ROC AUCs of the machine learning algorithms (i.e., MLP, RF, XGB) consistently show much better performance than the DC and vary between 70 and 90% (see Fig. 4). The introduction of the new class palliative care leads to a crucial decrease of sensitivity for the ICU class (varying between 7 and 31%), while sensitivities for the outpatient (i.e., discharge) and ward classes remain almost unchanged. Specificity for the ward class deteriorates for almost all algorithms, but remains constant for the outpatient (i.e., discharge) class and increases for the ICU class. In addition, ROC AUC providing an integrated view on sensitivity and specificity remains at a high level. The new class palliative care obtains a sensitivity score from 34 to 53%. Regarding precision, F1-scores and an algorithm preferred, the interpretations of Section 4.1 remain unchanged.

A detailed summary of the performance metrics is provided in Supplementary Table 2. A radar chart for a visual comparison of the different performance metrics discussed before is provided in Fig. 4. The confusion matrix and ROC AUC for the preferred XGB algorithm with four labels, an iterative Random Forest imputer, and the Charlson-Comorbidity Index (i.e., 4RC data processing) are presented in Supplementary Fig. 3.

## Discussion

Taking the different metrics into consideration, the performance of the base triage algorithm (TA) which is suggested as a guideline in Germany is significantly improved by an analytics-based adaptation: the extended triage algorithm (TAE). The AI-based algorithms and the integrated human-AI algorithm (ITA) perform similar, but significantly superior compared to the base triage algorithm (TA) and the extended triage algorithm (TAE). A major advantage of the integrated human-AI algorithm (ITA) is the high sensitivity with respect to the ICU category. The sensitivity for the ICU class is particularly important because especially ICU capacities have become scarce during the Covid-19 pandemic and the correct classification of critical care patients directly influences their well-being.

We find the human-AI interactive algorithm and the AI-based algorithms for superior performance. As the algorithms directly influence patients and medical staff in the emergency department not only a data-driven, but also ethical, usability, and implementation perspective are considered. Ethical considerations are mainly driven by the autonomy of the decision maker and the transparency of the algorithm, a basic characteristic in the XAI definition (see, e.g., [36, 45] or [6]). Human-AI interaction also contributes to XAI in healthcare [22]. The AI-based algorithms are non-transparent black-box models whereas the base triage algorithm and the extended triage algorithm (both being decision trees) are classified as transparent white-box models. The human-AI interactive model integrates both ideas and is partially transparent. Other than for the AI-based models, the physician, i.e., human approach, is the decision maker for the base and the extended triage algorithm. In case of human-AI algorithm, the decision is made interactively by the machine and the human being in a two-step approach.

Regarding usability and implementation, the decision trees, i.e., base, and extended triage algorithms, are preferable because decision support can already be provided through an easy-to-understand figure. For the AI-based and human-AI algorithms, elaborate implementation, and an interface to the hospital information system are essential. As there are different advances in many countries regarding digitalization in hospitals, e.g., the Hospital Future Act in Germany, the broad implementation of digital decision support tools will be made possible in near future.

The integrated human-AI algorithm performs similarly to the AI-based methods, but elucidates a higher sensitivity regarding the ICU category, it is partially transparent, and integrates the machine and the human being as decision makers. As implementation issues will be solved soon, the human-AI interactive algorithm is preferable. This result is not influenced by the distinct data preparation proceeding. The consideration of the pathway palliative care, which is controversial in Covid-19 triage (see, e.g., [28], is to be avoided from our data-driven perspective, and not only from ethical considerations. This conclusion is of particular importance in times of high ICU capacity utilization.

Our study builds upon an existing triage algorithm, a data set with more than 4,000 Covid-19 patients, and AI techniques. Due to the nature of the LEOSS registry, inpatients are significantly overrepresented, so the algorithm should not be applied to ambulatory care settings outside an emergency department. Limitations include the data quality due to missing values. By filling the data using the most frequent value, i.e., the simple imputer, a rather inaccurate approximation is assumed. Imputation using machine learning methods (RF, MLP) is more accurate in terms of the optimal solution, but the stopping criterion is not reached in certain cases. This can be attributed to the number of missing values. In addition, the LEOSS data builds upon predefined ranges regarding the categorization of demographic data and other parameters such as the blood counts. Thus, the scores, e.g., the CCI, are applied via an approximation, because the LEOSS ranges do not exactly match those of the respective scores.

In addition, the LEOSS dataset represents a European sample of infected individuals with a strong focus on German health care institutions. Varying prevalence rates, possible mutations or hygiene conditions in other countries could influence the result. Consequently, the results are assumed to be a representation of emergency departments in other European and developed countries in a comparable state of the pandemic, but further data is necessary to validate the algorithms for varying courses of the pandemic and emergency departments in non-developed countries. The algorithms concentrate on a specific emergency department setting, i.e., the classification of Covid-19 patients, but there is a certain ability to apply the algorithms to other emergency department settings, such as the classification of patients with viral infections in general, e.g., flu. The base triage algorithm was suggested during the first pandemic wave as a guideline in Germany, and we use the LEOSS data output at a rather early stage of the pandemic. Consequently, there might exist interdependencies, i.e., the outcomes in part of the LEOSS data could be influenced by the triaged outcomes using the base triage algorithm. On the other hand, based on LEOSS, we use the realized highest care unit of treatment, e.g., ICU, of each patient as ground truth label which is not necessarily defined based on the base triage algorithm.

## Conclusion

In this work, we evaluate the performance of Covid-19 triage algorithms in the emergency department and discuss the potential of integrating analytics, AI, XAI and human-AI interaction in detail. The results are based on a dataset with more than 4,000 PCR confirmed SARS-CoV-2 infected patients. Compared with existing papers, the size of our dataset significantly exceeds current literature.

We find that data-driven manipulation of the existing human-made base triage algorithm can improve the classification, but AI adaptations promise a superior performance. Comparing the AI methods with an integrated human-AI method, the algorithms are comparable in many performance metrics. But based on ethical AI considerations in terms of transparency, we suggest the use of the integrated human-AI algorithm. The data preparation process plays a subordinate role for the performance of the algorithms. The hypothetical consideration of the (meta-) pathway palliative care might be excluded from our data perspective for times when enough ICU beds are available. This finding is important for the ethical dimension on the broad triage discussion.

Our data-driven retrospective perspective lays the basis for a prospective evaluation of the human-AI algorithm and behavioral analyses in future research. Aspects such as information asymmetry in between humans and machines can be studied on using experiments in the emergency department.

## Supplementary Information

Below is the link to the electronic supplementary material.

Supplementary file1 (DOCX 259 kb)

## Data Availability

The data used in this study is not publicly available for the following reasons and can only be provided upon request. The data used is exclusively sensitive health care data, some of which is stored in a registry. Data protection declarations are available for the data.

## Footnotes

1 See https://khzg.de/
