# Supplementary material for: Covid-19 triage in the emergency department 2.0: how analytics and AI transform a human-made algorithm for the prediction of clinical pathways
Source: Health Care Manag Sci. 2023 Jul 10;26(3):412–29. doi: 10.1007/s10729-023-09647-2 (PMC10485125; doi:10.1007/s10729-023-09647-2)
Supplement: Supplementary file 1 — Supplementary file1 (DOCX 259 kb) [file 10729_2023_9647_MOESM1_ESM.docx]

**Supplementary Figures and Tables**


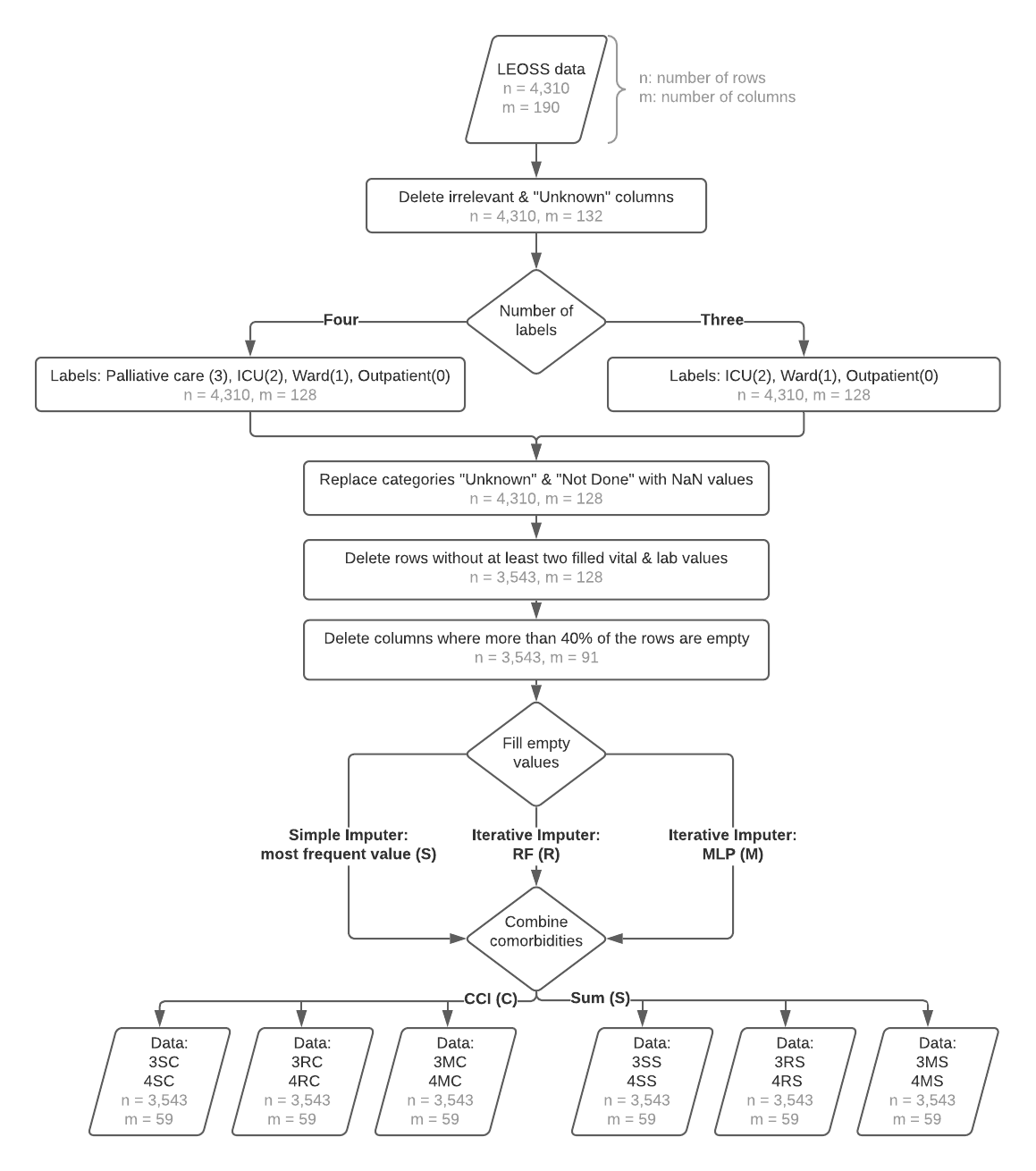


*Figure 1: Data preparation (every row in the dataset corresponds with a single patient, the columns denote the features)*


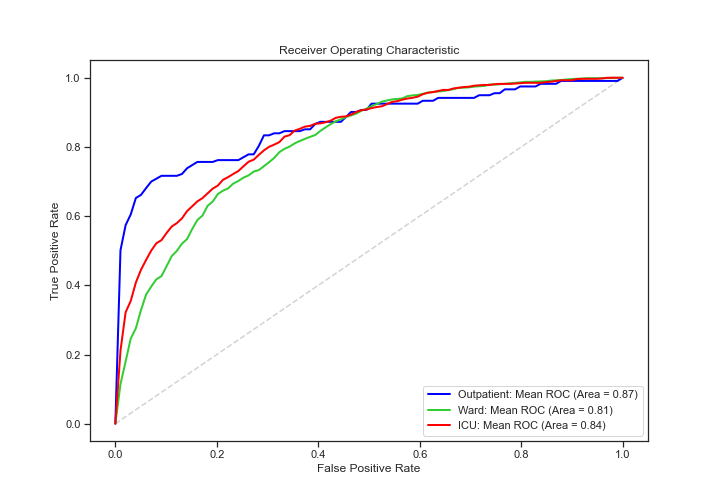

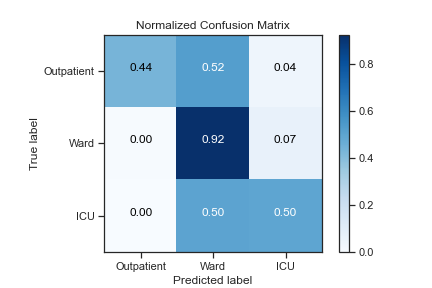


*Figure 2: Receiver operating characteristic (left hand side) and confusion matrix (right hand side) for the XGB Classifier (3MC).*


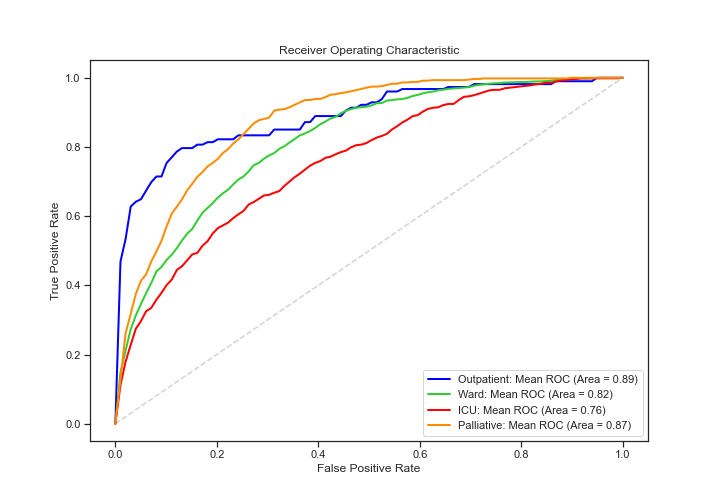

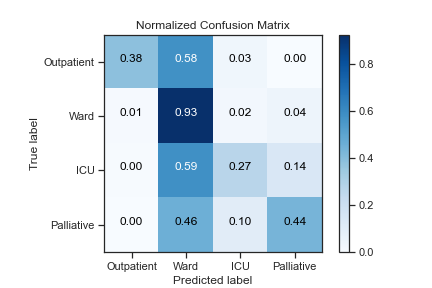


*Figure 3: Receiver operating characteristic (left hand side) and confusion matrix (right hand side) for the XGB Classifier (4RC)*

*Table 1: Evaluation metrics for the different algorithms (3 labels)*

| Accuracy | | | Recall | | | Precision | | | Specificity | | |
| --- | --- | --- | --- | --- | --- | --- | --- | --- | --- | --- | --- |
|  |  | Total | Outpatient | Ward | ICU | Outpatient | Ward | ICU | Outpatient | Ward | ICU |
| TA | 3RC | *0.27* | *0.84* | *0.14* | *0.53* | *0.06* | *0.81* | *0.36* | *0.54* | *0.92* | *0.65* |
|  | 3RS | *0.28* | *0.84* | *0.15* | *0.53* | *0.06* | *0.82* | *0.37* | *0.54* | *0.92* | *0.66* |
|  | 3MC | *0.28* | *0.65* | *0.16* | *0.54* | *0.05* | *0.79* | *0.36* | *0.56* | *0.90* | *0.64* |
|  | 3MS | *0.29* | *0.65* | *0.17* | *0.54* | *0.05* | *0.81* | *0.37* | *0.56* | *0.91* | *0.65* |
|  | 3SC | *0.27* | *0.58* | *0.15* | *0.52* | *0.04* | *0.77* | *0.35* | *0.55* | *0.89* | *0.64* |
|  | 3SS | *0.28* | *0.58* | *0.16* | *0.52* | *0.04* | *0.78* | *0.36* | *0.55* | *0.89* | *0.65* |
| TAE | 3RC | *0.50* | *0.73* | *0.53* | *0.40* | *0.10* | *0.71* | *0.50* | *0.75* | *0.52* | *0.85* |
|  | 3RS | *0.50* | *0.73* | *0.54* | *0.38* | *0.10* | *0.71* | *0.50* | *0.75* | *0.50* | *0.86* |
|  | 3MC | *0.51* | *0.69* | *0.53* | *0.43* | *0.10* | *0.72* | *0.49* | *0.77* | *0.53* | *0.83* |
|  | 3MS | *0.51* | *0.69* | *0.53* | *0.42* | *0.10* | *0.71* | *0.49* | *0.77* | *0.52* | *0.83* |
|  | 3SC | *0.48* | *0.68* | *0.53* | *0.32* | *0.08* | *0.70* | *0.47* | *0.72* | *0.49* | *0.87* |
|  | 3SS | *0.48* | *0.68* | *0.54* | *0.31* | *0.08* | *0.70* | *0.47* | *0.72* | *0.48* | *0.87* |
| DC | 3RC | *0.04* | *1.00* | *0.00* | *0.00* | *0.03* | *0.00* | *0.00* | *0.00* | *1.00* | *1.00* |
|  | 3RS | *0.04* | *1.00* | *0.00* | *0.00* | *0.03* | *0.00* | *0.00* | *0.00* | *1.00* | *1.00* |
|  | 3MC | *0.04* | *1.00* | *0.00* | *0.00* | *0.03* | *0.00* | *0.00* | *0.00* | *1.00* | *1.00* |
|  | 3MS | *0.04* | *1.00* | *0.00* | *0.00* | *0.03* | *0.00* | *0.00* | *0.00* | *1.00* | *1.00* |
|  | 3SC | *0.04* | *1.00* | *0.00* | *0.00* | *0.03* | *0.00* | *0.00* | *0.00* | *1.00* | *1.00* |
|  | 3SS | *0.04* | *1.00* | *0.00* | *0.00* | *0.03* | *0.00* | *0.00* | *0.00* | *1.00* | *1.00* |
| RF | 3RC | *0.77* | *0.39* | *0.94* | *0.39* | *0.65* | *0.78* | *0.76* | *0.99* | *0.39* | *0.95* |
|  | 3RS | *0.77* | *0.40* | *0.94* | *0.39* | *0.64* | *0.78* | *0.76* | *0.99* | *0.40* | *0.95* |
|  | 3MC | *0.78* | *0.44* | *0.94* | *0.40* | *0.75* | *0.78* | *0.76* | *0.99* | *0.41* | *0.95* |
|  | 3MS | *0.78* | *0.38* | *0.94* | *0.41* | *0.70* | *0.78* | *0.76* | *0.99* | *0.41* | *0.95* |
|  | 3SC | *0.74* | *0.37* | *0.93* | *0.30* | *0.64* | *0.76* | *0.65* | *0.99* | *0.33* | *0.94* |
|  | 3SS | *0.74* | *0.36* | *0.93* | *0.31* | *0.65* | *0.76* | *0.65* | *0.99* | *0.33* | *0.94* |
| XGB | 3RC | *0.78* | *0.44* | *0.93* | *0.47* | *0.68* | *0.80* | *0.73* | *0.99* | *0.47* | *0.94* |
|  | 3RS | *0.78* | *0.43* | *0.92* | *0.47* | *0.69* | *0.80* | *0.73* | *0.99* | *0.48* | *0.93* |
|  | 3MC | *0.79* | *0.44* | *0.92* | *0.50* | *0.82* | *0.80* | *0.73* | *1.00* | *0.49* | *0.93* |
|  | 3MS | *0.78* | *0.40* | *0.92* | *0.49* | *0.77* | *0.80* | *0.72* | *1.00* | *0.48* | *0.93* |
|  | 3SC | *0.74* | *0.40* | *0.91* | *0.38* | *0.58* | *0.77* | *0.62* | *0.99* | *0.40* | *0.91* |
|  | 3SS | *0.74* | *0.40* | *0.90* | *0.38* | *0.60* | *0.77* | *0.61* | *0.99* | *0.41* | *0.91* |
| MLP | 3RC | *0.76* | *0.45* | *0.88* | *0.50* | *0.54* | *0.80* | *0.65* | *0.99* | *0.51* | *0.90* |
|  | 3RS | *0.77* | *0.42* | *0.89* | *0.50* | *0.58* | *0.80* | *0.66* | *0.99* | *0.50* | *0.91* |
|  | 3MC | *0.78* | *0.42* | *0.90* | *0.52* | *0.70* | *0.81* | *0.68* | *0.99* | *0.51* | *0.91* |
|  | 3MS | *0.78* | *0.40* | *0.89* | *0.52* | *0.68* | *0.81* | *0.67* | *0.99* | *0.51* | *0.90* |
|  | 3SC | *0.75* | *0.48* | *0.88* | *0.44* | *0.50* | *0.79* | *0.61* | *0.98* | *0.47* | *0.89* |
|  | 3SS | *0.75* | *0.44* | *0.88* | *0.45* | *0.47* | *0.79* | *0.62* | *0.98* | *0.47* | *0.90* |
| ITA | 3RC | *0.73* | *0.60* | *0.72* | *0.69* | *0.37* | *0.84* | *0.53* | *0.96* | *0.70* | *0.77* |
|  | 3RS | *0.72* | *0.63* | *0.76* | *0.63* | *0.36* | *0.83* | *0.56* | *0.96* | *0.65* | *0.82* |
|  | 3MC | *0.72* | *0.62* | *0.72* | *0.72* | *0.50* | *0.85* | *0.53* | *0.98* | *0.72* | *0.76* |
|  | 3MS | *0.73* | *0.63* | *0.76* | *0.67* | *0.43* | *0.84* | *0.56* | *0.97* | *0.68* | *0.81* |
|  | 3SC | *0.68* | *0.53* | *0.72* | *0.62* | *0.36* | *0.82* | *0.49* | *0.97* | *0.65* | *0.76* |
|  | 3SS | *0.70* | *0.59* | *0.76* | *0.57* | *0.31* | *0.82* | *0.53* | *0.95* | *0.63* | *0.81* |

|  |  | ROC AUC | | | | F1 | | |
| --- | --- | --- | --- | --- | --- | --- | --- | --- |
|  |  | Total | Outpatient | Ward | ICU | Outpatient | Ward | ICU |
| TA | 3RC | *-* | *-* | *-* | *-* | *0.11* | *0.24* | *0.43* |
|  | 3RS | *-* | *-* | *-* | *-* | *0.11* | *0.26* | *0.44* |
|  | 3MC | *-* | *-* | *-* | *-* | *0.09* | *0.27* | *0.43* |
|  | 3MS | ***-*** | ***-*** | ***-*** | ***-*** | *0.09* | *0.28* | *0.44* |
|  | 3SC | *-* | *-* | *-* | *-* | *0.08* | *0.26* | *0.42* |
|  | 3SS | *-* | *-* | *-* | *-* | *0.08* | *0.27* | *0.43* |
| TAE | 3RC | *-* | *-* | *-* | *-* | *0.17* | *0.61* | *0.44* |
|  | 3RS | *-* | *-* | *-* | *-* | *0.17* | *0.61* | *0.43* |
|  | 3MC | ***-*** | ***-*** | ***-*** | ***-*** | *0.17* | *0.61* | *0.46* |
|  | 3MS | ***-*** | ***-*** | ***-*** | ***-*** | *0.17* | *0.61* | *0.45* |
|  | 3SC | *-* | *-* | *-* | *-* | *0.15* | *0.61* | *0.38* |
|  | 3SS | *-* | *-* | *-* | *-* | *0.15* | *0.61* | *0.37* |
| DC | 3RC | *0.50* | *0.50* | *0.50* | *0.50* | *0.07* | *0.00* | *0.00* |
|  | 3RS | *0.50* | *0.50* | *0.50* | *0.50* | *0.07* | *0.00* | *0.00* |
|  | 3MC | *0.50* | *0.50* | *0.50* | *0.50* | *0.07* | *0.00* | *0.00* |
|  | 3MS | *0.50* | *0.50* | *0.50* | *0.50* | *0.07* | *0.00* | *0.00* |
|  | 3SC | *0.50* | *0.50* | *0.50* | *0.50* | *0.07* | *0.00* | *0.00* |
|  | 3SS | *0.50* | *0.50* | *0.50* | *0.50* | *0.07* | *0.00* | *0.00* |
| RF | 3RC | *0.83* | *0.87* | *0.80* | *0.82* | *0.48* | *0.85* | *0.51* |
|  | 3RS | *0.83* | *0.87* | *0.80* | *0.82* | *0.49* | *0.85* | *0.51* |
|  | 3MC | *0.83* | *0.86* | *0.80* | *0.83* | *0.55* | *0.86* | *0.53* |
|  | 3MS | *0.83* | *0.86* | *0.80* | *0.83* | *0.49* | *0.85* | *0.53* |
|  | 3SC | *0.80* | *0.86* | *0.76* | *0.76* | *0.47* | *0.84* | *0.41* |
|  | 3SS | *0.79* | *0.85* | *0.76* | *0.77* | *0.47* | *0.83* | *0.42* |
| XGB | 3RC | *0.83* | *0.87* | *0.80* | *0.83* | *0.53* | *0.86* | *0.57* |
|  | 3RS | *0.83* | *0.87* | *0.81* | *0.83* | *0.53* | *0.86* | *0.57* |
|  | 3MC | *0.84* | *0.87* | *0.81* | *0.84* | *0.57* | *0.86* | *0.59* |
|  | 3MS | *0.84* | *0.86* | *0.81* | *0.84* | *0.52* | *0.86* | *0.58* |
|  | 3SC | *0.80* | *0.85* | *0.77* | *0.77* | *0.47* | *0.83* | *0.47* |
|  | 3SS | *0.80* | *0.85* | *0.77* | *0.78* | *0.48* | *0.83* | *0.47* |
| MLP | 3RC | *0.83* | *0.88* | *0.79* | *0.81* | *0.49* | *0.84* | *0.57* |
|  | 3RS | *0.83* | *0.87* | *0.79* | *0.82* | *0.49* | *0.84* | *0.57* |
|  | 3MC | *0.83* | *0.87* | *0.80* | *0.83* | *0.53* | *0.85* | *0.58* |
|  | 3MS | *0.83* | *0.87* | *0.80* | *0.83* | *0.51* | *0.85* | *0.59* |
|  | 3SC | *0.81* | *0.86* | *0.77* | *0.79* | *0.49* | *0.83* | *0.51* |
|  | 3SS | *0.81* | *0.86* | *0.78* | *0.79* | *0.45* | *0.83* | *0.52* |
| ITA | 3RC | *-* | *-* | *-* | *-* | *0.46* | *0.77* | *0.60* |
|  | 3RS | *-* | *-* | *-* | *-* | *0.46* | *0.79* | *0.59* |
|  | 3MC | *-* | *-* | *-* | *-* | *0.55* | *0.78* | *0.61* |
|  | 3MS | ***-*** | ***-*** | ***-*** | ***-*** | *0.51* | *0.80* | *0.61* |
|  | 3SC | *-* | *-* | *-* | *-* | *0.43* | *0.77* | *0.55* |
|  | 3SS | *-* | *-* | *-* | *-* | *0.41* | *0.79* | *0.55* |

*Table 2: Evaluation metrics for the different algorithms (4 labels)*

|  |  |  | Recall | | | | Precision | | | | Specificity | | | |
| --- | --- | --- | --- | --- | --- | --- | --- | --- | --- | --- | --- | --- | --- | --- |
|  |  | Total | Outpatient | Ward | ICU | Palliative | Outpatient | Ward | ICU | Palliative | Outpatient | Ward | ICU | Palliative |
| TAE | 4RC | *0.44* | *0.77* | *0.54* | *0.10* | *0.39* | *0.09* | *0.65* | *0.26* | *0.46* | *0.74* | *0.52* | *0.94* | *0.91* |
|  | 4RS | *0.45* | *0.77* | *0.54* | *0.09* | *0.39* | *0.09* | *0.65* | *0.25* | *0.48* | *0.74* | *0.51* | *0.94* | *0.91* |
|  | 4MC | *0.46* | *0.75* | *0.54* | *0.10* | *0.47* | *0.10* | *0.67* | *0.24* | *0.47* | *0.76* | *0.55* | *0.93* | *0.90* |
|  | 4MS | *0.46* | *0.75* | *0.54* | *0.08* | *0.46* | *0.10* | *0.66* | *0.21* | *0.48* | *0.76* | *0.54* | *0.93* | *0.90* |
|  | 4SC | *0.44* | *0.70* | *0.55* | *0.08* | *0.34* | *0.08* | *0.65* | *0.22* | *0.46* | *0.72* | *0.52* | *0.94* | *0.92* |
|  | 4SS | *0.44* | *0.70* | *0.56* | *0.07* | *0.34* | *0.08* | *0.65* | *0.20* | *0.47* | *0.72* | *0.51* | *0.94* | *0.92* |
| DC | 4RC | *0.03* | *1.00* | *0.00* | *0.00* | *0.00* | *0.03* | *0.00* | *0.00* | *0.00* | *0.00* | *1.00* | *1.00* | *1.00* |
|  | 4RS | *0.03* | *1.00* | *0.00* | *0.00* | *0.00* | *0.03* | *0.00* | *0.00* | *0.00* | *0.00* | *1.00* | *1.00* | *1.00* |
|  | 4MC | *0.03* | *1.00* | *0.00* | *0.00* | *0.00* | *0.03* | *0.00* | *0.00* | *0.00* | *0.00* | *1.00* | *1.00* | *1.00* |
|  | 4MS | *0.03* | *1.00* | *0.00* | *0.00* | *0.00* | *0.03* | *0.00* | *0.00* | *0.00* | *0.00* | *1.00* | *1.00* | *1.00* |
|  | 4SC | *0.03* | *1.00* | *0.00* | *0.00* | *0.00* | *0.03* | *0.00* | *0.00* | *0.00* | *0.00* | *1.00* | *1.00* | *1.00* |
|  | 4SS | *0.03* | *1.00* | *0.00* | *0.00* | *0.00* | *0.03* | *0.00* | *0.00* | *0.00* | *0.00* | *1.00* | *1.00* | *1.00* |
| RF | 4RC | *0.71* | *0.39* | *0.94* | *0.23* | *0.41* | *0.63* | *0.73* | *0.61* | *0.62* | *0.99* | *0.42* | *0.97* | *0.95* |
|  | 4RS | *0.70* | *0.38* | *0.94* | *0.20* | *0.38* | *0.62* | *0.72* | *0.59* | *0.61* | *0.99* | *0.39* | *0.97* | *0.95* |
|  | 4MC | *0.69* | *0.37* | *0.93* | *0.18* | *0.42* | *0.57* | *0.72* | *0.50* | *0.64* | *0.99* | *0.40* | *0.96* | *0.95* |
|  | 4MS | *0.69* | *0.35* | *0.94* | *0.15* | *0.41* | *0.53* | *0.71* | *0.49* | *0.64* | *0.99* | *0.38* | *0.97* | *0.95* |
|  | 4SC | *0.67* | *0.41* | *0.92* | *0.14* | *0.34* | *0.65* | *0.69* | *0.48* | *0.57* | *0.99* | *0.33* | *0.97* | *0.95* |
|  | 4SS | *0.66* | *0.38* | *0.93* | *0.10* | *0.30* | *0.63* | *0.69* | *0.39* | *0.55* | *0.99* | *0.30* | *0.97* | *0.95* |
| XGB | 4RC | *0.71* | *0.38* | *0.93* | *0.27* | *0.44* | *0.68* | *0.74* | *0.60* | *0.58* | *0.99* | *0.47* | *0.96* | *0.94* |
|  | 4RS | *0.71* | *0.38* | *0.93* | *0.26* | *0.42* | *0.67* | *0.74* | *0.56* | *0.58* | *0.99* | *0.46* | *0.96* | *0.94* |
|  | 4MC | *0.70* | *0.37* | *0.92* | *0.22* | *0.48* | *0.65* | *0.74* | *0.50* | *0.61* | *0.99* | *0.47* | *0.95* | *0.94* |
|  | 4MS | *0.70* | *0.35* | *0.93* | *0.21* | *0.46* | *0.65* | *0.74* | *0.48* | *0.62* | *0.99* | *0.46* | *0.95* | *0.94* |
|  | 4SC | *0.67* | *0.42* | *0.91* | *0.16* | *0.37* | *0.61* | *0.71* | *0.43* | *0.53* | *0.99* | *0.40* | *0.95* | *0.93* |
|  | 4SS | *0.67* | *0.44* | *0.91* | *0.16* | *0.36* | *0.59* | *0.72* | *0.43* | *0.53* | *0.99* | *0.40* | *0.95* | *0.93* |
| MLP | 4RC | *0.69* | *0.42* | *0.86* | *0.31* | *0.49* | *0.59* | *0.76* | *0.45* | *0.55* | *0.99* | *0.55* | *0.92* | *0.92* |
|  | 4RS | *0.69* | *0.44* | *0.86* | *0.31* | *0.48* | *0.62* | *0.76* | *0.45* | *0.54* | *0.99* | *0.56* | *0.92* | *0.92* |
|  | 4MC | *0.70* | *0.43* | *0.89* | *0.27* | *0.53* | *0.64* | *0.76* | *0.46* | *0.60* | *0.99* | *0.53* | *0.93* | *0.93* |
|  | 4MS | *0.70* | *0.43* | *0.89* | *0.28* | *0.52* | *0.67* | *0.76* | *0.46* | *0.59* | *0.99* | *0.54* | *0.93* | *0.93* |
|  | 4SC | *0.67* | *0.49* | *0.86* | *0.22* | *0.47* | *0.46* | *0.74* | *0.42* | *0.54* | *0.98* | *0.50* | *0.93* | *0.92* |
|  | 4SS | *0.67* | *0.45* | *0.87* | *0.20* | *0.45* | *0.49* | *0.74* | *0.38* | *0.53* | *0.98* | *0.49* | *0.93* | *0.92* |

|  |  | ROC AUC | | | | | F1 | | | |
| --- | --- | --- | --- | --- | --- | --- | --- | --- | --- | --- |
|  |  | Total | Outpatient | Ward | ICU | Palliative | Outpatient | Ward | ICU | Palliative |
| TAE | 4RC | *-* | *-* | *-* | *-* | *-* | *0.17* | *0.59* | *0.14* | *0.42* |
|  | 4RS | *-* | *-* | *-* | *-* | *-* | *0.17* | *0.59* | *0.13* | *0.43* |
|  | 4MC | *-* | *-* | *-* | *-* | *-* | *0.17* | *0.60* | *0.14* | *0.47* |
|  | 4MS | *-* | *-* | *-* | *-* | *-* | *0.17* | *0.60* | *0.12* | *0.47* |
|  | 4SC | *-* | *-* | *-* | *-* | *-* | *0.14* | *0.60* | *0.11* | *0.39* |
|  | 4SS | *-* | *-* | *-* | *-* | *-* | *0.14* | *0.60* | *0.10* | *0.39* |
| DC | 4RC | *0.50* | *0.50* | *0.50* | *0.50* | *0.50* | *0.06* | *0.00* | *0.00* | *0.00* |
|  | 4RS | *0.50* | *0.50* | *0.50* | *0.50* | *0.50* | *0.06* | *0.00* | *0.00* | *0.00* |
|  | 4MC | *0.50* | *0.50* | *0.50* | *0.50* | *0.50* | *0.06* | *0.00* | *0.00* | *0.00* |
|  | 4MS | *0.50* | *0.50* | *0.50* | *0.50* | *0.50* | *0.06* | *0.00* | *0.00* | *0.00* |
|  | 4SC | *0.50* | *0.50* | *0.50* | *0.50* | *0.50* | *0.06* | *0.00* | *0.00* | *0.00* |
|  | 4SS | *0.50* | *0.50* | *0.50* | *0.50* | *0.50* | *0.06* | *0.00* | *0.00* | *0.00* |
| RF | 4RC | *0.84* | *0.90* | *0.82* | *0.76* | *0.87* | *0.48* | *0.82* | *0.33* | *0.50* |
|  | 4RS | *0.84* | *0.90* | *0.82* | *0.76* | *0.87* | *0.47* | *0.82* | *0.29* | *0.46* |
|  | 4MC | *0.83* | *0.89* | *0.81* | *0.75* | *0.88* | *0.45* | *0.81* | *0.26* | *0.51* |
|  | 4MS | *0.83* | *0.89* | *0.81* | *0.74* | *0.88* | *0.42* | *0.81* | *0.23* | *0.50* |
|  | 4SC | *0.80* | *0.87* | *0.77* | *0.70* | *0.84* | *0.50* | *0.79* | *0.22* | *0.43* |
|  | 4SS | *0.80* | *0.88* | *0.77* | *0.70* | *0.84* | *0.47* | *0.79* | *0.16* | *0.39* |
| XGB | 4RC | *0.84* | *0.89* | *0.82* | *0.76* | *0.87* | *0.49* | *0.83* | *0.37* | *0.50* |
|  | 4RS | *0.83* | *0.89* | *0.82* | *0.76* | *0.87* | *0.49* | *0.82* | *0.25* | *0.49* |
|  | 4MC | *0.83* | *0.88* | *0.81* | *0.75* | *0.88* | *0.47* | *0.82* | *0.30* | *0.54* |
|  | 4MS | *0.83* | *0.87* | *0.81* | *0.74* | *0.88* | *0.46* | *0.82* | *0.29* | *0.53* |
|  | 4SC | *0.80* | *0.86* | *0.78* | *0.70* | *0.85* | *0.50* | *0.80* | *0.23* | *0.44* |
|  | 4SS | *0.80* | *0.86* | *0.78* | *0.70* | *0.84* | *0.50* | *0.80* | *0.23* | *0.43* |
| MLP | 4RC | *0.82* | *0.89* | *0.80* | *0.73* | *0.86* | *0.49* | *0.81* | *0.36* | *0.52* |
|  | 4RS | *0.82* | *0.89* | *0.80* | *0.73* | *0.86* | *0.52* | *0.81* | *0.37* | *0.51* |
|  | 4MC | *0.83* | *0.89* | *0.81* | *0.74* | *0.88* | *0.51* | *0.82* | *0.34* | *0.56* |
|  | 4MS | *0.83* | *0.89* | *0.81* | *0.74* | *0.87* | *0.52* | *0.82* | *0.34* | *0.55* |
|  | 4SC | *0.81* | *0.88* | *0.79* | *0.71* | *0.85* | *0.47* | *0.80* | *0.29* | *0.50* |
|  | 4SS | *0.81* | *0.88* | *0.79* | *0.71* | *0.85* | *0.47* | *0.80* | *0.27* | *0.49* |
